# An exercise intervention to prevent falls in Parkinson’s: an economic evaluation

**DOI:** 10.1186/1472-6963-12-426

**Published:** 2012-11-23

**Authors:** Emily Fletcher, Victoria A Goodwin, Suzanne H Richards, John L Campbell, Rod S Taylor

**Affiliations:** 1 Primary Care Research Group, University of Exeter Medical School, Smeall Building, St Luke’s Campus, Magdalen Road, Exeter, EX1 2LU, UK; 2 PenCLAHRC, University of Exeter Medical School, Veysey Building, Salmon Pool Lane, Exeter, EX2 4SG, UK; 3 Institute of Health Services Research, University of Exeter Medical School, Veysey Building, Salmon Pool Lane, Exeter EX2 4SG, UK

**Keywords:** Economic evaluation, Cost effectiveness, Parkinson’s, Falls prevention, Rehabilitation

## Abstract

**Background:**

People with Parkinson’s (PwP) experience frequent and recurrent falls. As these falls may have devastating consequences, there is an urgent need to identify cost-effective interventions with the potential to reduce falls in PwP. The purpose of this economic evaluation is to compare the costs and cost-effectiveness of a targeted exercise programme versus usual care for PwP who were at risk of falling.

**Methods:**

One hundred and thirty participants were recruited through specialist clinics, primary care and Parkinson’s support groups and randomised to either an exercise intervention or usual care. Health and social care utilisation and health-related quality of life (EQ-5D) were assessed over the 20 weeks of the study (ten-week intervention period and ten-week follow up period), and these data were complete for 93 participants. Incremental cost per quality adjusted life year (QALY) was estimated. The uncertainty around costs and QALYs was represented using cost-effectiveness acceptability curves.

**Results:**

The mean cost of the intervention was £76 per participant. Although in direction of favour of exercise intervention, there was no statistically significant differences between groups in total healthcare (−£128, 95% CI: -734 to 478), combined health and social care costs (£-35, 95% CI: -817 to 746) or QALYs (0.03, 95% CI: -0.02 to 0.03) at 20 weeks. Nevertheless, exploration of the uncertainty surrounding these estimates suggests there is more than 80% probability that the exercise intervention is a cost-effective strategy relative to usual care.

**Conclusion:**

Whilst we found no difference between groups in total healthcare, total social care cost and QALYs, analyses indicate that there is high probability that the exercise intervention is cost-effective compared with usual care. These results require confirmation by larger trial-based economic evaluations and over the longer term.

## Background

Parkinson’s is a progressive neurodegenerative disorder that mainly affects older individuals. The prevalence rates of Parkinson’s are estimated at 1 per cent in people aged over 60 years and between 0.15 and 0.3 per cent in the general population with a mean age of onset in the mid sixties [1]. Cost of illness studies have shown that Parkinson’s is costly for individuals, the health-care system and society more broadly [2,3]. Hospitalisation and drug therapy account for more than sixty percent of the direct costs associated with Parkinson’s [4]. Annual UK expenditure is in excess of £1.4 billion taking account of health and social care, loss of productivity and informal care costs [5] with costs increasing as the condition progresses [6].

PwP experience frequent and recurrent falls. As many as 65 percent of fallers will experience an injury secondary to falling, of whom 33 percent will sustain a fracture [7]. Although cost of illness studies have not quantified the costs of falls and fall-related injuries for those with Parkinson’s, it estimated that as much as 75 percent of falls result in additional health care utilisation and associated costs [8]. Moreover, the fear of falling results in restriction of activities of daily living and can markedly compromise health-related quality of life (HRQoL) [9]. Any intervention that reduces falls in PwP might therefore be expected to have important impact on costs and HRQoL.

A systematic review of exercise interventions aimed at PwP reported a paucity of studies in relation to falls prevention, with no studies to date examining the costs or cost-effectiveness of any interventions aimed at preventing falls in PwP [10]. There have, however, been reviews of economic evaluations of fall prevention interventions targeting older people that indicate the cost effectiveness of exercise interventions [11], home safety modifications for those at high risk of falling and multi-factorial assessments with targeted interventions [12]. A pragmatic randomised controlled trial (GETuP) was recently undertaken to assess the effectiveness of a targeted exercise programme in PwP who have a history of falling [13]. Although no significant difference in the rate of falls was observed in those undertaking a 10 week exercise intervention compared with usual care controls, the exercise group did experience superior gains in Berg balance, Falls Efficacy Scale-International scores and recreational physical activity levels [13].

In the present paper we describe an economic evaluation undertaken alongside the GETuP trial. This economic evaluation aimed to assess the costs of the provision of an exercise intervention for the prevention of falls in PwP and whether this intervention was cost-effective in terms of quality adjusted life years (QALYs).

## Methods

Full details of the trial design, participants, interventions, and outcome assessments are described elsewhere [13]. Ethical approval was obtained from the Devon and Torbay Research Ethics committee (07/Q2102/8) and written informed consent was provided by all participants. In summary, participants were recruited through specialist clinics, primary care and Parkinson’s support groups in the South West of England between May 2007 and November 2008. A total of 130 participants with a diagnosis of Parkinson’s and self-reported history of two or more falls in the preceding 12 months were randomised to either participate in a ten-week group exercise programme with supplementary home exercises (intervention group, n=64) or to continue with usual care alone (control group, n=66). Both groups were assessed at baseline (prior to randomisation) and at 20 weeks (following ten-week intervention period plus a ten-week follow up period). The intervention comprised once weekly group exercise sessions, with twice weekly home strength and balance training exercises, commencing ten weeks after the baseline assessment. For each group, sessions were delivered in community settings, National Health Service physiotherapists, with experience of working with older people and those with Parkinson’s. The size of the groups was set at a maximum of six participants due to space restrictions at some venues, and also for reasons of safety and group management [14]. All participants received usual care that could include medical and medication management, physiotherapy (e.g., exercise, advice, provision of walking aids, gait training), occupational therapy (e.g., modification of home hazards, provisions of aids or adaptations) or speech therapy.

For the purposes of this economic analysis, we measured costs from the perspective of UK National Health Service and Personal Social Services. A cost utility analysis was performed: both groups were analysed for their differences in total costs compared with differences in QALYs. The study was conducted and reported in accordance with health economics reporting guidelines [15].

### Assessment of health outcome

The health status of participants was assessed using the EQ-5D (or EuroQol), a generic and validated measure of choice for which reliable UK population preference values are available [16]. The EQ-5D measures health on five domains (mobility, self-care, usual activities, pain–discomfort, and anxiety–depression), and the scores were combined to generate a single utility value. Published utility values for the EQ-5D range from 1.00 for the ‘best health’ state to −0.594 for the ‘worst health’ state, where a score of 0 is regarded as equivalent to death, and scores less than 0 are considered as ‘worse than death’ [17]. Participants completed the EQ-5D at baseline and 20 weeks. During this follow-up, participants were assessed twice: first following a ten-week group exercise intervention (post-intervention), and then again at 10 weeks follow-up. Participants who died during the study were registered as zero in utility terms for the assessment period from when the death occurred. We calculated the number of QALYs gained or lost over the 20 weeks of follow up using the area under the curve, and we assumed a linear change in utility between measurement points [18,19].

### Quantifying use of resources

The time and grade of physiotherapists involved in the delivery of the exercise intervention was recorded via records kept by the research team together with the cost of venue hire, equipment costs and travel costs incurred by physiotherapists and participants. Travel costs were calculated on distance travelled from participant’s home (or physiotherapist’s base) to the exercise venue based on postcodes [20]. We calculated the average intervention cost per participant assuming all participants randomised to the intervention group (n=64) attended all of the exercise classes [13].

At each follow up visit, participants reported their use of Parkinson’s medication (type and dose). The number of hospital contacts, primary-care and social service contacts were obtained for each participant using routine databases from local hospital Trusts, general practices and Devon County Council Social Services.

### Costs

In order to translate medication and health and social service resource use into monetary values, unit costs or prices were applied [15,21,22]. Resources were valued using both local or national costs and prices (Table 1). Staff costs included indirect overheads (the costs of support services such as human resources, finance, and estates needed to carry out the service’s main functions) and building capital (the costs assigned to intervention space). All unit costs were calculated for the financial years 2008/9.

**Table 1:** Unit costs for health and social care resource use

**Resource item**^**†**^	**Cost**	**Basis of estimate**
**Primary care**		
General Practitioner (GP) surgery appointments	£36.00	An 11.7 minute consultation
General Practitioner (GP) home visits	£58.00	A 23.4 minute visit
Practice nurse (PN) surgery appointments	£11.00	A consultation
Community/district nurse (CN) visits	£26.00	A home visit
**Hospital**-**based care**		
*Acute hospital bed days	£213.20	A bed day of inpatient rehabilitation for an elderly person (£205 inflated by 1.04)
Day admissions	£138.00	A day care service attendance for an elderly person
Accident & Emergency (A&E) attendances	£111.00	An A&E treatment
Minor Injury Unit (MIU) attendances	£35.00	An attendance at a non 24-hour A&E department/Casualty department
Outpatient consultant appointments	£55.00	A first-attendance appointment
	£71.00	A follow-up attendance appointment
^Parkinson’s Disease specialist nurse (PDNS) appointments	£15.00	A 15 minute consultation (using ‘Nurse advanced [includes lead specialist, clinical nurse specialist, senior specialist])
^Parkinson’s Disease specialist nurse (PDNS) visits	£16.66	A 25 minute home visit (using ‘Nurse advanced [includes lead specialist, clinical nurse specialist, senior specialist]) at £40/hour
**Social care**		
Social worker/community care manager assessments	£414.00	An hour of face-to-face contact = £138 Average length of contact (from DCC info provided to QAQoL) = 3hours
Hours of home care (per hour)	£19.30	One hour of local authority organized home care
Hours of day care (per hour)	£11.66	A session = £35
		Estimated 3 hours per session
Days of residential care (per day)	£136.86	Local authority care package for a short-term resident week = £958
Days of nursing care (per day)	£93.71	Private care package cost for a short-term resident week = £656

† The unit costs of resource items were identified using 2008 cost data from the Personal Social Services Research Unit (Curtis, 2008).

* No 2008 cost data were available for acute hospital bed days, so the PSSRU 2007 cost was inflated for this item (Curtis, 2007).

^ No unit cost data were available for PDNS care, so the PSSRU 2008 costs for a ‘Nurse advanced (includes lead specialist, clinical nurse specialist, senior specialist) were used, as these relate to the same NHS pay band (Band 7) as PDNS, quoted by NICE 2006.

### Data analysis and statistical methods

Intervention and control groups were compared at follow up, based on randomised allocation. Health and social care contacts were analysed using logistic and Poisson regression and expressed as rate and risk ratios and their associated 95% confidence intervals (CIs). Although costs were not normally distributed, analyses compared the mean costs in the two groups using the student’s *t*-test with ordinary least squares regression used to adjust the between group comparison for baseline costs. Parametric statistical analysis allowed us to make inferences about mean costs [23]. Ninety five percent CIs for mean difference in costs were calculated using the non-parametric bias corrected bootstrapping method (1000 replications) [15]. As there were no differences in inferences between unadjusted (for baseline values) and adjusted models, we report only unadjusted results here.

Cost-effectiveness is concerned with the joint difference in costs and outcome between interventions and was assessed over the 20 week period (i.e. ten-week intervention period combined with the ten-week follow-up period) through the calculation of an incremental cost-effectiveness ratio (ICER) [24]. The ICER is the ratio of differential average costs of the intervention and control group to the differential QALYs.

Repeat re-sampling from the costs and QALY data (bootstrapping) was used to generate a distribution of mean costs and QALYs for the two participant groups [25]. These distributions are used to calculate the probability that intervention or control is the optimal choice, subject to a range of possible maximum monetary values (ceiling ratio, λ) that a decision-maker might be willing to pay for an increase in QALYs. These probabilities are presented as cost-effectiveness acceptability curves [19] and a judgement made cost-effectiveness on the basis of maximum willingness to pay threshold of £20,000 per QALY, (lower boundary of acceptable cost effectiveness of the National Institute for Health and Clinical Excellence) [15,26]. These curves incorporate the uncertainty that exists around the estimates of mean costs and effects as a result of sampling variation with uncertainty regarding the maximum cost-effectiveness ratio that a decision-maker would consider acceptable.

The impact of missing QALY and cost data were assessed by comparing the baseline characteristics of participants who had missing data with those participants who had full economic data. In addition we undertook sensitivity analysis to examine the impact of the cost-effectiveness analysis of missing QALY and cost data using two approaches to data imputation i.e. last value carried forward and assumed ‘missing at random’ using multiple imputation by chained equations [27].

The time horizon of the analysis did not require discounting of QALYs or costs. All analyses were undertaken in Stata v1 (Stata Corp, College Station, Texas).

## Results

Of the 130 participants who were randomised, 37 were excluded from the economic analysis due to missing resource-use or EQ-5D data. Thus, 93 participants had complete data on total resource costs and QALYs over the 20 week follow-up period and were entered into the economic evaluation (48 intervention group participants, 45 control group participants). A comparison of baseline characteristics revealed some significant differences between those included in the economic evaluation and those for whom data were missing (Table 2). Those participants with missing data were significantly more likely to be male and had significantly higher healthcare costs during the baseline data collection period (the ten weeks immediately prior to the commencement of the intervention). However, there was no difference in missing data between intervention and control.

**Table 2:** Comparison of baseline characteristics for participants with missing and available economic data

	**Available (N=93)**	**Missing (N=37)**
**Age in years** mean (SD)	71.0 (8.8)	71.1 (7.7)
**Male** (%)	48 (52%)	26 (70%)
**Years since diagnosis of PD** mean (SD)	8.8 (6.5)	8.3 (6.2)
**Hoehn and Yahr stage** Mean (SD)	2.5 (0.9)	2.6 (0.9)
**Living arrangements n** (%)		
Alone	21 (23%)	12 (32%)
With partner	68 (73%)	24 (65%)
With family/friends	2 (2%)	1 (3%)
Residential home	2 (2%)	0 (0%)
**Parkinson**’**s medication** n (%)		
LCT	88 (95%)	36 (97%)
DRA	46 (49%)	18 (49%)
MOAI	16 (17%)	6 (16%)
**Co**-**morbidity n** (%)		
Orthopaedic	31 (33%)	15 (41%)
Cardiac	30 (32%)	16 (43%)
**EQ**-**5D** mean (SD)	0.67 (0.23)	0.68 (0.25)
**Total healthcare costs in previous 10 weeks** (£) mean (SD)+	142 (227)	768 (481)

+ Primary and secondary care costs - data only available in 115 individuals (90/25).

### Exercise intervention costs

The total cost to deliver the 10 week exercise intervention was £4,883 (venue hire: £448; physiotherapist time: £3,900; physiotherapist travel: £335; equipment: £200) and corresponded to an average cost of £76 per participant. This average cost assumed that all participants attended the intervention and was based on an average of approximately 4 participants per exercise group. However, if the maximum group size of six participants had been achieved this average cost could be reduced to £54 per participant. The mean participant travel cost in attending the intervention sessions was £20 (standard deviation: £2).

### Health and social care service resource use

Table 3 details the mean healthcare utilisation across both groups. Use of medication, and of primary and secondary care services was compared and no significant differences were observed.

**Table 3:** Health care resource utilisation at 20 weeks

	**Intervention (n=48)**	**Control (n=45)**	**Effect size (95% CI), P-value**
**Parkinson’s medication**			
LCT n/N (%)	42/45 (91%)	38/41 (93%)	Relative risk −0.96 (0.86to 1.08), 0.524
GA n/N (%)	1/46 (2%)	3/42 (7%)	Relative risk 0.32 (0.03 to 2.96), 0.315
AC n/N (%)	0/47 (0%)	1/44 (2%)	Not calculable
CTI n/N (%)	1/47 (2%)	3/42 (7%)	Relative risk 0.31 (0.03 to 2.89), 0.306
DRA n/N (%)	19/45 (42%)	17/42 (40%)	Relative risk 1.04 (0.63 to 1.72), 0.869
MOBI n/N (%)	4/46 (9%)	5/43 (12%)	Relative risk 0.75 (0.21 to 2.60), 0.648
**Primary care contacts**			
GP appointments, mean (SD)	2.19 (3.05)	1.96 (2.58)	Rate ratio 1.12 (0.84 to 1.48), 0.438
GP home visits, mean (SD)	0.08 (0.28)	0.09 (0.29)	Rate ratio 0.92 (0.23 to 3.66), 0.902
PN appointments., mean (SD)	0.83 (1.17)	0.84 (1.42)	Rate ratio 0.99 (0.63 to 1.56), 0.984
CN home visits, mean (SD)	0.04 (0.19)	0.23 (0.99)	Rate ratio 0.15 (0.02 to 1.28), 0.084
**Acute healthcare contacts**			
Hospitalisations, mean (SD)	0.06 (0.24)	0.33 (0.74)	Rate ratio 0.19 (0.05 to 0.65), 0.008
Inpatient bed days, mean (SD)*	0.50 (3.18)	1.51 (6.30)	Mean difference −1.01 (−3.04 to 1.02), 0.326
Day cases, mean (SD)	0.02 (0.14)	0.11 (0.38)	Rate ratio 0.19 (0.02 to 1.60), 0.126
A&E attendances, mean (SD)	0.10 (0.37)	0.16 (0.56)	Rate ratio 0.67 (0.21 to 2.11), 0.493
Outpatient consultant			
Appointments, mean (SD)	2.25 (2.14)	2.69 (2.79)	Rate ratio 0.84 (0.65 to 1.08), 0.178
PDNS appointments, mean (SD)	0 (0)	0.02 (0.15)	Not calculable
PDNS home visits, mean (SD)	0 (0)	0 (0)	Not calculable
**Social care contacts**			
Social/community care assessments	0.17 (0.52)	0.04 (0.21)	Rate ratio 3.74 (0.80 to 17.63), 0.095
Home care received, n/N (%)	2/45 (4%)	1/48 (2%)	Relative risk 1.88 (0.17 to 19.97), 0.60
Hours of home care, mean (SD) †	9.15 (45.9)	2.00 (13.42)	Mean difference 7.14 (−7.00 to 21.29), 0.318
Day care received, n/N (%)	1/47 (2%)	0/45 (0%)	Not calculable
Hours of day care, mean (SD)**	0.42 (2.91)	0 (0)	Mean difference 0.42 (−0.44 to 1.29), 0.331
Residential care received, n/N (%)	1/44 (2%)	0/48 (0%)	Not calculable
Hours of residential care, mean (SD)	0 (0)	0.44 (2.98)	Mean difference −0.44 (1.30 to 0.41), 0.304
Nursing care relieved, n/N (%)	1/44 (2%) 0 (0)	0/48 (0%) 0.44 (2.98)	Not calculable
			Mean difference −0.44 (1.30 to 0.41), 0.304
Hours of nursing care, mean††			

* Number of bed days in those with a hospital admission.

† Number of hours of home care in those with a home care visit.

** Number of residential care days in those in residential care.

†† Number of nursing care days in those who received nurse care.

### Health and social care service costs

Table 4 details the cost analyses. Mean total acute healthcare cost per participant in the intervention group was £263, on average £389 per participant lower than for controls. This cost difference was mainly due to a lower cost of inpatient bed days in the intervention group. In contrast, social care costs were higher by £97 per participant for the intervention arm due to higher levels of social care assessment and home care. As shown in Table 4 none of the between group differences in total or subtotal costs (e.g. medication, acute healthcare contacts) were statistically significant.

**Table 4:** Healthcare costs at 20 weeks

	**Intervention Mean (SD), median N=48**	**Control Mean (SD), median N=45**	**Mean difference (95% CI) P-value**
**Parkinson’s medications (£)**			
Mean (SD)	836 (994), 579	707 (812), 331	129 (−490 to 233), 0.50
**Acute healthcare (£)**			
Inpatient bed days, mean (SD)	107 (678), 0	322 (1339), 0	−215 (−643 to 212), 0.324
Day case, mean (SD)	3 (20), 0	15 (53), 0	−12 (−30 to 5), 0.156
A&E attendances, mean (SD)	12 (41), 0	17 (62), 0	−6 (−28 to 17), 0.616
Outpatient consultant appointments, mean (SD)	142 (19), 126	169 (26), 162	−28 (−92 to 36), 0.398
PDNS appointments, mean (SD)	0 (0), 0	1 (2), 0	1 (−1 to 1), 0.321
PDNS home visits, mean (SD)	0 (0), 0	0 (0), 0	Not calculable
Total acute healthcare, mean (SD)	263 (749), 126	524 (1429), 126	−389 (−741 to 190), 0.27
**Primary healthcare contacts (£)**			
GP appointments, mean (SD)	79 (110), 36	70 (93), 36	8 (−31 to 48), 0.677
GP home visit, mean (SD)	5 (16), 0	5 (17), 0	0 (−7 to 6), 0.923
Parkinson nurse appointments, mean (SD)	9 (13), 0	9 (15), 0	0 (−5 to 6), 0.901
Community nurse home visit, mean (SD)	1 (4), 0	3 (20), 0	−3 (−9 to 3), 0.324
Total primary healthcare, mean (SD)	93 (116), 69	88 (101), 62	5 (−38 to 49), 0.80
**Social care contacts (£)**			
Social/community care assessments, mean (SD)	70 (217), 0	18 (86), 0	52 (−15 to 119), 0.126
Hours of home care, mean (SD)	177 (886), 0	39 (259), 0	138 (−129 to 405), 0.312
Hours of day care, mean (SD)	5 (34), 0	0 (0), 0	5 (−5 to 15), 0.324
Days of residential care, mean (SD)	0 (0), 0	61 (408), 0	−61 (−173 to 52), 0.290
Days of nursing care, mean (SD)	0 (0), 0	42 (279), 0	−42 (−123 to 40), 0.318
Total social care, mean (SD)	252 (1023), 0	159 (753), 0	92 (−268 to 453), 0.62

PDNS: Parkinson specialist nurse.

### HRQoL, QALYs and cost-effectiveness analysis

**Figure 1:**
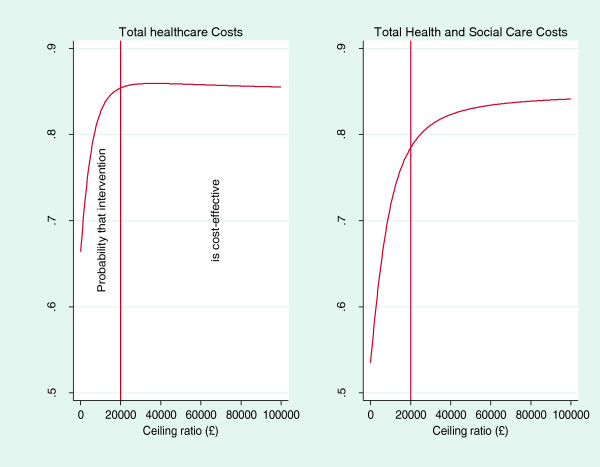
Cost effectiveness acceptability curves for intervention based on QALY gains based on total healthcare costs and combined total health and social care costs.

The EQ-5D scores used to calculate QALYs are summarised in Table 5. There was no statistically significant gain in average QALYs from baseline to follow up for intervention group participants compared to controls (0.03, 95% CI: -0.02 to 0.08)., The ICER for total healthcare costs was approximately -£4,900 per QALY and the ICER for combined total healthcare and social care costs was approximately -£1,400 per QALY (Table 6). Although both ICERs indicate that the intervention is likely to be dominant compared to control i.e. cheaper and more effective, the between group differences in costs and QALYs were not statistically significant. Figure 1 illustrates the uncertainty associated with the ICERs and demonstrates that, for values of £0 to £100,000, the intervention has a higher probability of being cost-effective than control. The probability of intervention being more cost effective at £20,000 was 85% when considering total healthcare costs and 81% for combined healthcare and social care costs. Conclusions did not change with the use of imputed full sample data. Approximate ICERs for the full imputed sample were -£15,500/QALY (last observation carried forward) and -£6,000/QALY (multiple imputation) for total healthcare costs and -£3,900/QALY (last observation carried forward) and -£3,100/QALY (multiple imputation) for combined social and healthcare costs.

**Table 5:** EQ-5D results at baseline, 10 and 20 weeks

	**Intervention (n=48)**	**Control (n=45)**	Effect size (95% CI)*, P-value
Baseline	0.70 (0.22)	0.65 (0.23)	
10 weeks	0.66 (0.29)	0.66 (0.23)	−0.04 (−0.12 to 0.05), P=0.395
20 weeks	0.74 (0.29)	0.62 (0.30)	0.08 (−0.03 to 0.19), P=0.131

*Adjusted for baseline EQ-5D score.

**Table 6:** QALYs and incremental cost effectiveness ratio at trial follow-up

	**Intervention Mean (SD) N=48**	**Control Mean (SD) N=45**	**Mean difference Mean (95% CI), P-value**	**ICER (£) Cost per QALY**
**QALYs**	0.40 (0.13)	0.37 (0.12)	0.03 (−0.02 to 0.08), 0.32	
**Total healthcare costs (£)**	1198 (1192)	1320 (1676)	−128 (−734 to 478). 0.68	−4,885
**Total health and social care costs (£)**	1444 (1953)	1479 (1982)	−35 (−817 to 746), 0.93	−1,358

## Discussion

### Study findings

This study found no statistically significant between group differences in total health care costs, combined health and social care costs or QALYs at 20 weeks follow-up. However, the 95% CIs around these estimates are wide, suggesting that our analysis may be underpowered to detect such differences. The presentation of cost-effectiveness acceptability curves are important within this context, demonstrating that there was over an 80% probability that the exercise intervention is a more cost effective option at willingness to pay of £20,000/QALY. Assuming participation by all intervention participants in the exercise classes (n=64), the average healthcare cost of the 10 week exercise intervention was £76 per participant, although this could potentially be reduced to £54 per participant if the maximum participant:staff ratio of 6:1 had been achieved.

### Comparisons with previous literature

Although no studies have examined the costs and cost-effectiveness of exercise programmes aimed at preventing falls in PwP, a small number have examined this issue in community-dwelling elderly populations [28-30]. Three studies have consistently demonstrated exercise training to be cost-effective compared with usual care, and have reported incremental cost effectiveness ratios of $US 1,538 (1993 US dollars), $AUZ 4,986 (1997 Auz dollars) and $NZ 1,803 (1998 NZ dollars) per fall prevented respectively. However, there are limitations in using these studies to inform current UK healthcare policy on exercise in the management of Parkinson’s. Firstly, all were conducted in an elderly community-dwelling sample of participants and therefore have uncertain generalisability to PwP. Secondly, given the varying international patterns of healthcare utilisation, the results may not directly applicable to UK. Finally, the scope of resource use costed in these studies was limited, focusing on intervention costs and costs associated with the prevention of falls rather than the full extent of healthcare costs.

Comparison with other research involving exercise to reduce falls reveals a number of studies that have produced a mean cost per participant for a 1-year exercise intervention in elderly populations. Three previous studies have reported the mean costs of exercise interventions aimed at reducing falls in elderly populations. Robertson et al. (2001) reported a cost per participant for a home-based exercise intervention delivered by a nurse to individual participants for three months (followed by a three-month maintenance period) of between $NZ 173 (£83) and $NZ 432 (£208) per patient [31,32]. Timonen et al. (2008) reported a cost of 568 EUR (£583.54) per participant for a ten-week, group-based exercise program for frail elderly women after discharge from hospital following acute illness [33], and Rizzo et al. (1996) reported $USD 905 (£582) per participant for a one-off, individually-targeted falls prevention program for an elderly community population [28]. The cost of the one-off ten week group exercise intervention therefore appears to be relatively inexpensive to exercise interventions applied in previous evaluations of community-dwelling elderly populations.

### Strengths and weaknesses

This economic evaluation was set within a randomised controlled trial. The aim was to examine the full scope of health and social care resource use of participants on the impact of the intervention for PwP at risk of falling. Although every effort was made to collect all aspects of healthcare resource use data for all randomised participants, difficulties were experienced in accounting for some aspects of community-based primary care services. This was due to a lack of routine recording of contacts with physiotherapy, occupational therapy, speech therapy, dietetics and community hospitals. As a result, contacts with these services were omitted from the final analyses. In addition, where it was not possible to collect full data from aspects of health and social care, via routine records, such as contacts with community hospital services, speech therapists and dietetics, these participants were excluded from the final analyses. Given the older age of the participants in this study, we did not deem it appropriate to peruse a fully societal perspective and seek costs associated with lost productivity.

Participants with missing economic data at follow up were found at have higher healthcare costs at entry to this trial suggesting they have been more severe in their disease and that our findings are subject to attrition bias. However, those participants with missing and complete data appeared balanced in terms of other indicators of disease severity as according to Hoehn and Yahr stage. Nevertheless this loss of data certainly reduced overall statistical power to detect differences in HRQoL and costs outcomes at follow up. Our findings were robust to sensitivity analyses using two methods of data imputation.

Cost-effectiveness is influenced by the time horizon over which the analysis is evaluated. Shorter periods of evaluation are often associated with less-favourable cost-effectiveness, suggesting that perspectives motivated by short-term gains may not see as much benefit from the interventions that prevent longer-term morbidity and premature mortality. The economic evaluation of the GETuP trial examined resource use and cost over the ten weeks of the intervention and a further ten weeks during follow-up. Extended evaluation beyond this time would provide evidence for any longer-term benefits of the exercise intervention. As discussed earlier, there are no such extrapolation studies in the area of exercise rehabilitation for Parkinson’s. However, decision modelling has been applied to more expensive drug therapies in order demonstrate their potential cost-effectiveness over the lifetime of the PwP [34]. Falls among PwP are associated with carer burden [35], although we did not quantify the impact on carers, in terms of HRQoL or lost productivity. Carer burden of is a potentially important area that deserves be considered in future economic studies.

Reporting an incremental cost per QALY allows policy makers to make comparisons between resource allocations for different medical conditions. It is recognised however, that the EQ-5D, as a generic and not a disease-specific instrument, may lack sufficient sensitivity to detect changes in HRQoL. The issue of the potential insensitivity of generic HRQoL measures was highlighted by a recent systematic review of economic evaluations of exercise interventions for reducing falls in elderly populations [6]. Future research is needed to better understand the relative sensitivity of disease-specific HRQoL and generic measures such as the EQ-5D in the Parkinson’s populations. A recently published study has developed a mapping approach to link the Parkinson’s disease Questionnaire (PDQ-39) to the EQ-5D [36].

## Conclusions

We know of no prior evidence on the cost-effectiveness of an exercise-based intervention for PwP who are frequent fallers. This study has shown the exercise intervention to be relatively inexpensive and therefore likely to be cost-effective if a small health gain can be shown. Although results of the present study are supportive of the cost-effectiveness of an exercise intervention targeting a reduction in falls, we recognise that this study may be underpowered to make a definitive conclusion in terms of the differences in costs and QALY between groups. Larger scale economic evaluation studies are therefore needed in order to affirm the findings of this study before a definitive policy recommendation can be made. In order to minimise bias, future economic evaluations should to be undertaken with the context of an RCT, preferably with long term (≥ 12-months) follow-up of outcome and costs. We are aware of ongoing research in this area, such as two Australian trials of an eight-week and six-month falls prevention exercise intervention respectively for PwP [37,38].

## Competing interests

The authors declared that they have no competing interests.

## Authors’ contributions

EF and VG carried out data collection. VG, SR, JC and RT were involved in the conception and design of the study. RT acquired funding and undertook statistical analysis. All authors contributed to the draft of the manuscript and have read and approved the final version.

## Pre-publication history

The pre-publication history for this paper can be accessed here:

http://www.biomedcentral.com/1472-6963/12/426/prepub
